# Development of a short-term nutritional risk prediction model for hepatocellular carcinoma patients: a retrospective cohort study

**DOI:** 10.1038/s41598-024-54456-4

**Published:** 2024-02-16

**Authors:** Jiaxiang Yu, Kim Lam Soh, Liping He, Pengpeng Wang, Yingjuan Cao

**Affiliations:** 1 Department of Nursing, Universiti Putra Malaysia, 43400 Serdang, Selangor Malaysia; 2 Nursing Department, Qilu Hospital, Shandong University, Jinan, 250012 China

**Keywords:** Hepatocellular carcinoma, Nutritional risk, Short-term prognosis, Predictive model, Cancer, Disease prevention, Patient education, Quality of life

## Abstract

Malnutrition in patients is associated with reduced tolerance to treatment-related side effects and higher risks of complications, directly impacting patient prognosis. Consequently, a pressing requirement exists for the development of uncomplicated yet efficient screening methods to detect patients at heightened nutritional risk. The aim of this study was to formulate a concise nutritional risk prediction model for prompt assessment by oncology medical personnel, facilitating the effective identification of hepatocellular carcinoma patients at an elevated nutritional risk. Retrospective cohort data were collected from hepatocellular carcinoma patients who met the study's inclusion and exclusion criteria between March 2021 and April 2022. The patients were categorized into two groups: a normal nutrition group and a malnutrition group based on body composition assessments. Subsequently, the collected data were analyzed, and predictive models were constructed, followed by simplification. A total of 220 hepatocellular carcinoma patients were included in this study, and the final model incorporated four predictive factors: age, tumor diameter, TNM stage, and anemia. The area under the ROC curve for the short-term nutritional risk prediction model was 0.990 [95% CI (0.966–0.998)]. Further simplification of the scoring rule resulted in an area under the ROC curve of 0.986 [95% CI (0.961, 0.997)]. The developed model provides a rapid and efficient approach to assess the short-term nutritional risk of hepatocellular carcinoma patients. With easily accessible and swift indicators, the model can identify patients with potential nutritional risk more effectively and timely.

## Introduction

Primary liver cancer, of which hepatocellular carcinoma (HCC) accounts for over 85–90% of cases, ranks as the second primary cause of death globally^1^. Notably, China has a disproportionately high prevalence of liver cancer cases, with nearly 50% of cases globally, and demonstrates a mortality and morbidity rate exceeding the global average^2–4^.

Nutritional risk refers to the possibility of nutrition-related factors leading to adverse patient outcomes^5^. Findings suggest that a significant proportion of liver cancer patients, approximately 51.11%, are prone to nutritional risk^6^. Inadequately nourished patients typically present reduced tolerance to treatment-related side effects and an elevated likelihood of complications. It is worth noting that the extent of malnourishment has a strong correlation with patient prognosis^7^.

The nutritional risk of cancer patients has become increasingly recognized, highlighting the importance of assessing the patient's nutritional status to guide their care and treatment. Presently, risk prediction predominantly involves Nutritional Risk Screening (NRS-2002) and Patient-Generated Subjective Global Assessment (PG-SGA). Nonetheless, the accuracy of NRS-2002 is evaluated by relevant indicators. Meanwhile, subjective judgment concerning PG-SGA may be adversely impacted by factors such as diminished appetite and patient fatigue among those with liver cancer. Although PG-SGA holds high specificity and sensitivity, systematic training is also required to facilitate screening^8^.

The generalizability of previous research conclusions in predicting short-term nutritional risk among HCC patients is open to debate. Medical professionals may provide varying nutritional risk assessments for patients due to variations in work experience and other factors. Furthermore, there exists a disparity in scientific and efficient methods for evaluating nutritional risk among HCC patients.

This study aims to develop a concise nutritional risk prediction model for patients suffering from hepatocellular carcinoma. The creation of such a model will not only serve as a reliable tool for the early detection of nutritional deficiencies but also aid in the provision of early nutritional intervention, treatment, and nursing for patients.

## Methods

### Study design and population

**Figure 1:**
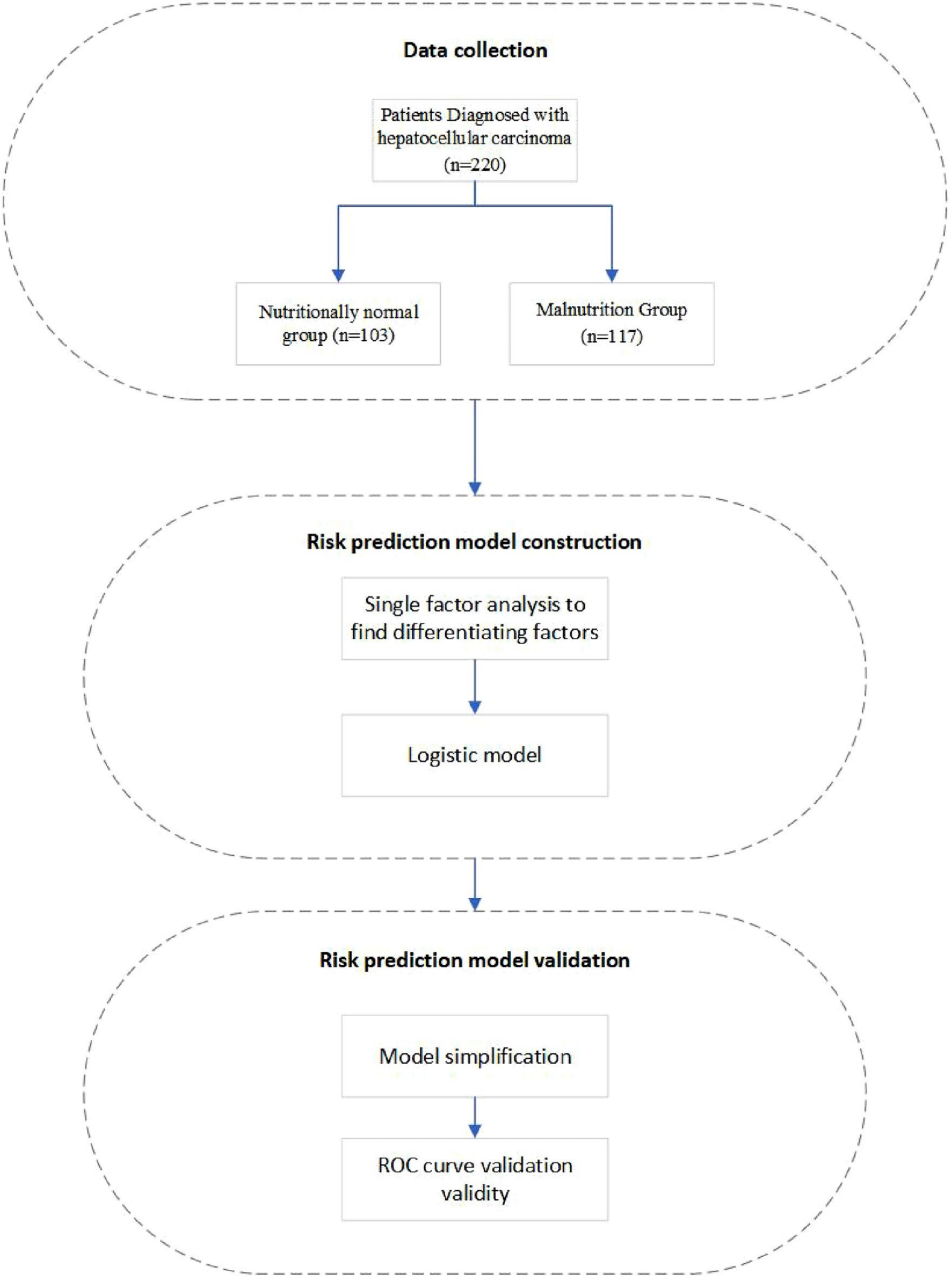
Study flow chart.

For this retrospective study, clinical data from patients diagnosed with hepatocellular carcinoma (HCC) were analyzed. The patients were admitted to the tumor ward of the hospital between March 2021 and April 2022. Study design flow chart are provided in Fig. 1.

### Ethical statement

The research has obtained ethical approval from the In-Hospital Ethics Committee of the Institute in accordance with the guidelines and regulations governing research involving human subjects. The study was approved by the Ethics Committee of Guangxi Medical University in Guangxi, China (Approval Number: No. 2022-KT-桂卫-005). All subjects signed the informed consent.

All personal information and data collected during the study will be handled with strict confidentiality and solely utilized for research purposes.

### Inclusion criteria

Participants in the study were selected based on the following inclusion criteria: (1) Diagnosis of hepatocellular carcinoma as per the ‘Diagnosis and Treatment Standards for Primary Liver Cancer (2020 Edition)’, ensuring alignment with the latest clinical guidelines. (2) Patients classified as having stage II or III liver cancer, representing a specific segment of the disease progression. (3) A minimum hospitalization duration of two weeks, allowing for adequate observation and data collection. (4) Availability of complete clinical data for each patient, ensuring a comprehensive analysis and robustness of the study findings.

### Exclusion criteria

The study adhered to specific exclusion criteria to ensure the integrity and focus of the research. Patients were excluded from the study if they met any of the following conditions: (1) Presence of other malignancies or distant metastases, to maintain a cohort specifically dealing with isolated hepatocellular carcinoma. (2) Diagnosis of severe mental illness, considering the potential impact on the reliability of self-reported data and adherence to treatment protocols. (3) Classification as Child–Pugh class C, in order to concentrate on patients with less severe liver impairment and avoid confounding factors associated with advanced liver disease.

A cohort of 220 individuals diagnosed with hepatocellular carcinoma (HCC) was thoughtfully chosen based on stringent inclusion and exclusion criteria. Following ESPEN guidelines, we employed dual-energy X-ray absorptiometry (DXA) to ascertain the presence of sarcopenia, a pivotal element within malnutrition assessment. Subsequently, patients were categorized into malnourished and normotrophic groups using DXA, a widely adopted, cost-effective, and highly reliable methodology. DXA, despite necessitating low doses of ionizing radiation, facilitates the measurement of body composition (BC) at both regional and systemic levels, employing a three-compartment model encompassing bone mineral content (BMC), fat mass (FM), and lean mass (LM). Subsequent diagnosis of malnutrition or normal nutrition relies on sex-specific AWGS DXA cutoffs (< 7.0 kg/m^2^ for men, < 5.4 kg/m^2^ for women) to delineate low muscle mass, and is confirmed through assessment by a certified nutritionist^9,10^.

Study variables included general patient information, such as gender, marital status, age, place of residence, and education. Additionally, disease data consisting of the number of masses, tumor diameter, stage, anemia, hepatitis B, adverse reactions, and Child–Pugh classification were also collected for analysis.

The Nutritional Risk Screening (NRS)-2002 was designed in 2002 by the European Society for Parenteral and Enteral Nutrition (ESPEN) to simplify the nutritional risk screening of hospitalized patients^5^. Since then, it has been recommended by the Chinese Society of Parenteral and Enteral Nutrition and Chinese Medical Association for nutritional risk assessment^11^. This method is swift and effective, requiring only three minutes to complete, and assigns a cumulative score based on the combination of impaired nutritional status and disease severity scores, for instance, one point for patients aged ≥ 70 years^12^. A cumulative score of 3 or higher indicates the presence of nutritional risk.

McCarthy et al.^13^ introduced the Patient-Generated Subjective Global Assessment-based SGA 2003, a widely accepted nutritional evaluation standard for cancer patients recognized by the American Dietitian Association and the Chinese Anti-Cancer Association^14,15^. This questionnaire is split into a self-assessment form and a medical staff scoring form. The patient self-assessment form covers weight, eating habits, symptoms, physical activities, and function. The medical staff scoring form contains information concerning the correlation between disease and nutritional needs, along with metabolic specifications and physical examination. The nutritional problem's severity becomes more apparent with higher scores.

## Results

This study enrolled a total of 220 patients, with a median age of 51 (42, 59) years, ranging from 27 to 72 years old, including 162 males and 58 females. Amongst them, 22 were unmarried, 155 were married, and 43 were divorced/widowed. The diameter of the tumors ranged from 3.29 cm to 16.03 cm, with a median diameter of 8.11 (6.94, 9.83) cm. The NRS-2002 score ranged from 0 to 7, with a median score of 4 (1, 5). Out of these patients, 77 had anemia, whereas 143 did not. Furthermore, 127 patients had viral hepatitis B, while 93 did not. According to the Child–Pugh grading, 130 cases were classified as grade A, and 90 cases were classified as grade B. Out of the total 220 patients, 117 (53.2%) were categorized in the malnutrition group, and 103 (46.8%) were categorized in the normal nutrition group.

### Univariate analysis was performed to investigate the short-term nutritional risk factors among patients diagnosed with hepatocellular carcinoma

Following the classification of patients into malnutrition and normal nutrition groups based on body composition analysis, comparative analysis revealed statistically significant differences in age, place of residence, tumor diameter, TNM stage, presence or absence of anemia, presence or absence of viral hepatitis B, NRS-2002, and PG-SGA between the two groups (all *P* values < 0.05) (Table 1).

**Table 1:** Univariate analysis of short-term nutritional risk factors in the perioperative period for liver cancer.

Variables	Malnutrition group (n = 117)	Nutritionally normal group (n = 103)	Statistical value	*P* value
Gender [n (%)]			0.067^b^	0.457
Male	87 (74.4)	75 (72.8)		
Female	30 (25.6)	28 (27.2)		
Marital status [n (%)]			4.385^b^	0.112
Unmarried	8 (6.8)	14 (13.6)		
Married	89 (76.1)	66 (64.1)		
Divorced/Widowed	20 (17.1)	23 (22.3)		
Age (years, $\overline{x} \pm s$)^a^	59 (56,62)	42 (36,46)	19.482^c^	0.000
Residence [n (%)]			13.606^b^	0.000
City	37 (31.6)	58 (56.3)		
Rural	80 (68.4)	45 (43.7)		
Education [n (%)]			3.816^b^	0.148
Junior high school and below	52 (44.4)	38 (36.9)		
High School/Secondary School	40 (34.2)	31 (30.1)		
College and above	25 (21.4)	34 (33.0)		
Number of lumps [n (%)]			0.374^b^	0.318
Single	74 (63.2)	61 (59.2)		
Multiple	43 (36.8)	42 (40.8)		
Tumor diameter (cm, $\overline{x} \pm s$)^a^	9.82 (8.39,11.19)	7.18 (6.53,7.96)	10.764^c^	0.000
TNM stage [n (%)]			14.281^b^	0.000
II	61 (52.1)	79 (76.7)		
III	56 (47.9)	24 (23.3)		
Anemia [n (%)]			29.121^b^	0.000
Yes	60 (51.3)	17 (16.5)		
No	57 (48.7)	86 (83.5)		
Viral hepatitis B [n (%)]			13.552^b^	0.000
Yes	81 (69.2)	46 (44.7)		
No	36 (30.8)	57 (55.3)		
Child–Pugh classification [n (%)]			0.056^b^	0.460
A	70 (59.8)	60 (58.3)		
B	47 (40.2)	43 (41.7)		
NRS-2002^a^	5 (4,6)	1 (0,2)	21.399^c^	0.000
PG-SGA			163.008^b^	0.000
A	2 (1.7)	89 (86.4)		
B	97 (82.9)	14 (13.6)		
C	18 (15.4)	0 (0)		

^a^M (*P*_25_, *P*_75_); ^b^*c*^*2*^ value; ^c^*Z* value.

### Establish and simplify the short-term nutritional risk prediction model for individuals diagnosed with hepatocellular carcinoma

Based on the results of Table 1, significant factors identified through the univariate analysis, including age, tumor diameter, TNM stage, anemia, viral hepatitis B status, and place of residence, were selected to be different in the two groups.

Out of 220 patients, 198 (90%) underwent active anti-tumor therapy. Specifically, 41 patients received LR, 8 underwent RFA, and 10 underwent LT. Additionally, 98 patients received TACE, 13 received TACE + RFA, 7 underwent LR + RFA, and 21 received systemic anti-tumor therapy as their initial treatment. In Best Supportive Care (BSC) for hepatocellular carcinoma, a holistic and tailored approach is employed to alleviate symptoms and enhance quality of life. This strategy includes meticulous symptom management using medications for pain, nausea, and fatigue; comprehensive psychological support through counseling and therapy; targeted management of liver-related complications such as ascites and jaundice; palliative procedures for symptomatic relief; and extensive social support and care coordination. Detailed results are provided in Table 2.

**Table 2:** Treatment of patients with different TNM staging levels, n (%).

Treatments	TNM staging		
	Total (n = 220)	II (n = 140)	III (n = 80)
LR	41 (18.6)	20 (14.3)	21 (26.2)
RFA	8 (3.6)	7 (5)	1 (1.2)
LT	10 (4.5)	6 (4.3)	4 (5)
TACE	98 (44.5)	65 (46.4)	33 (41.3)
TACE + RFA	13 (5.9)	11 (7.9)	2 (2.5)
LR + RFA	7 (3.2)	4 (2.9)	3 (3.8)
Systemic antitumor therapy	21 (9.5)	13 (9.3)	8 (10)
BSC	22 (10)	14 (10)	8 (10)

*LR* Liver resection; *RFA* Radiofrequency ablation; *LT* Liver transplantation; *TACE* Transcatheter arterial chemoembolization; *BSC* Best supportive care.

### Critical cutoff values for age and tumor diameter in predicting nutritional risk

The patients' nutritional status is categorized as either malnutrition or normal nutrition. In order to improve the practical application of the prediction model and promptly assess patients' nutritional risk, age and tumor diameter were converted into binary variables. Optimal cutoff values for these continuous variables were determined using the ROC curve function of Med-Calc 22.009 (https://www.medcalc.org/) software, along with the calculation of the Youden index for predicting nutritional risk. Detailed results are provided in Table 3.

**Table 3:** Optimal cut-off points and Youden index of age and tumor diameter for predicting short-term nutritional risk changes in patients with hepatocellular carcinoma.

Predictor variable	Best cutoff	Youden index	Sensitivity	Specificity
Age	51	0.8586	0.8974	0.9612
Tumor diameter	8.11	0.6578	0.8034	0.8544

Following the identification of cut-off values in Table 3, continuous variables were converted into dichotomous variables according to the following criteria: age ≤ 51 years = 0, age > 51 years = 1; tumor diameter ≤ 8.11 cm = 0, tumor diameter > 8.11 cm = 1; TNM stage: stage II = 0, stage III = 1; absence of anemia = 0, presence of anemia = 1; rural residence = 0, urban residence = 1; absence of hepatitis B = 0, presence of hepatitis B = 1. Subsequently, logistic regression analysis using the entry method was performed to establish a prediction model. The results of parameter estimation and testing for this regression yielded statistically significant results for four independent variables (age, tumor diameter, TNM stage, and anemia) (*P* < 0.05). The Hosmer–Lemeshow goodness of fit test was used to validate the model, yielding a result of *χ*^2^ = 3.647 and *P* = 0.887. The final logistic regression equation was *Logit(p)* = -5.616 + 6.495*age + 3.629*tumor diameter + 2.034*TNM stage + 1.897*anemia, as indicated in Table 4.

**Table 4:** Short-term nutritional risk prediction model for patients with hepatocellular carcinoma.

Predictor variable	Regression coefficients	Standard error	Wald value	*OR value*	95%CI		*P value*
					Lower limit	Upper limit	
Constant	− 5.616	1.100	26.047	–	–	–	–
Age	6.495	1.089	35.552	661.574	78.239	5594.112	0.000
Tumor diameter	3.629	0.929	15.269	37.678	6.103	232.608	0.000
TNM staging	2.034	0.861	5.584	7.642	1.415	41.286	0.018
Anemia	1.897	0.799	5.639	6.663	1.393	31.880	0.018
Residence	− 0.757	0.742	1.041	0.469	0.110	2.009	0.308
Hepatitis B	0.817	0.729	1.256	2.263	0.543	9.437	0.262

**Figure 2:**
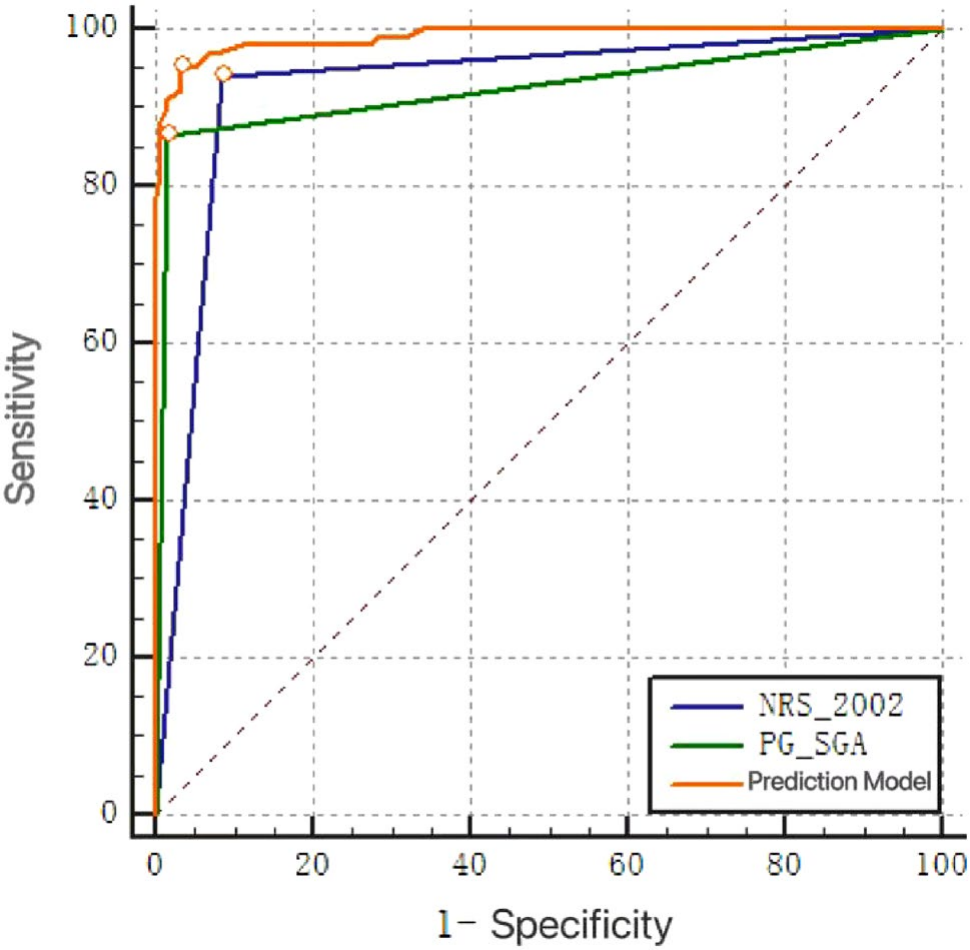
ROC curve of the short-term nutritional risk prediction model and other two scoring criteria in patients with hepatocellular carcinoma.

Figure 2 displays the ROC curves generated using the logistic regression model developed in this study in conjunction with the NRS-2002 and PG-SGA scales. The calculated AUCs for these variables were 0.990, 0.928, and 0.923, respectively.

The regression coefficients obtained for age, tumor diameter, TNM stage, and anemia, which have predictive significance in the logistic regression model, were reduced to simplified integers. Specifically, these values correspond to 6, 4, 2, and 2 points, respectively, within the score prediction model. According to this model, age was assigned 0 points for individuals aged 51 years or younger, and 6 points for individuals older than 51 years. Tumor diameter was assigned 0 points for values less than or equal to 8.11 cm, and 4 points for values greater than 8.11 cm. For TNM stage, 0 points were assigned for stage II and 2 points for stage III. The absence of anemia received 0 points, while a diagnosis of anemia yielded 2 points.

**Figure 3:**
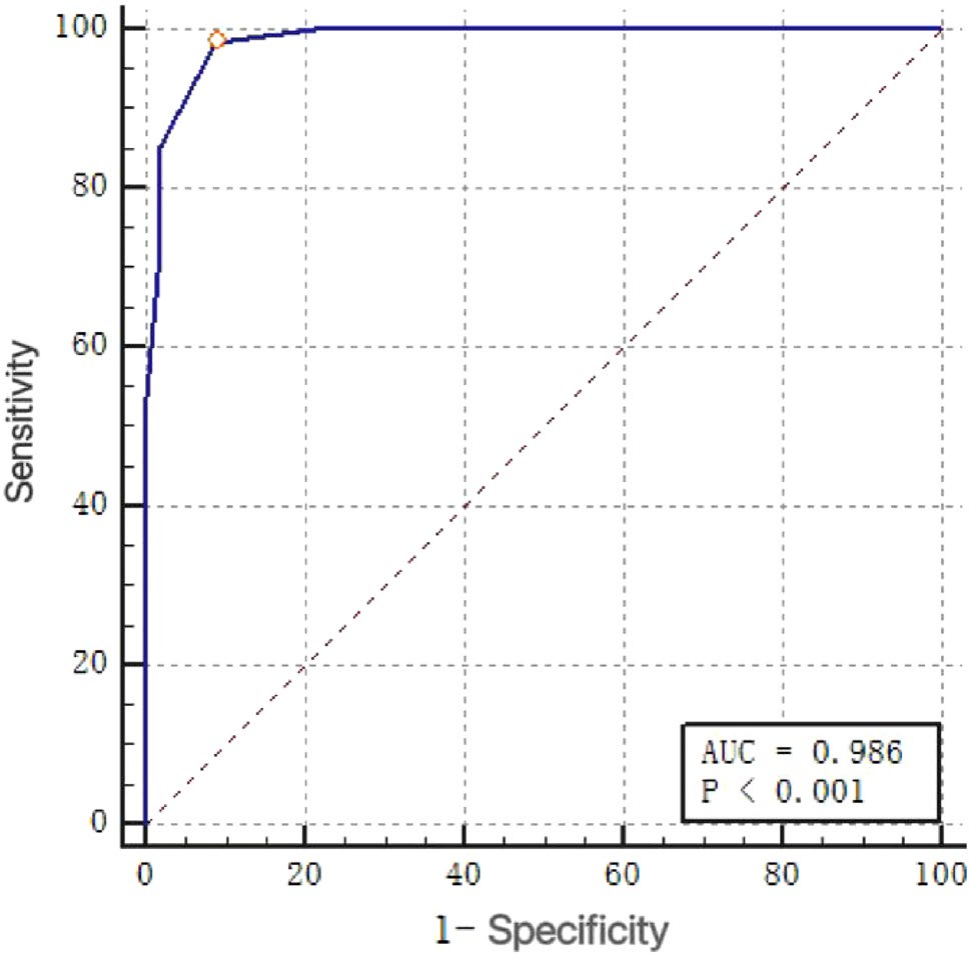
The ROC curve of the simplified scoring rule of the short-term nutritional risk prediction model for hepatocellular carcinoma patients.

The simplified new prediction model can be expressed as *Logit(n)* = 6*age + 4*tumor diameter + 2*tumor stage + 2*anemia, with a score range of 0 to 14 points. The AUC for this simplified model is 0.986, as illustrated in Fig. 3 using the generated ROC curve.

According to Med-Calc software's calculations, a cut-off score of > 4.

## Discussion

The incidence of malnutrition in patients with liver cancer is generally high, with the rate varying depending on the location and type of the tumor. Studies have attributed progressive malnutrition in these patients to low food intake and excessive energy consumption, as the tumor has shifted its metabolic pathway and increased metabolic rate^16^. Although there exist many methods and indicators for assessing malnutrition in patients, no single “gold standard” has been identified for those with hepatocellular carcinoma^17^.

The NRS-2002 score is a nutritional screening tool proposed and recommended by ESPEN in 2003. It is the only evidence-based nutritional screening tool based on 128 randomized controlled studies, and is simple and fast to use^18^. However, this scoring method does not improve the situation of patients with hepatocellular carcinoma, as they are often bedridden and unable to measure their weight or have edema, severe ascites, etc. Furthermore, the NRS-2002 is not applicable when unclear patients (*e.g.*, with hepatic encephalopathy) are unable to answer the assessor's questions.

PG-SGA is a standard developed for the nutritional evaluation of tumor patients, which is beneficial for the comprehensive assessment of patients' nutritional status. It can accurately assess tumor-related symptoms, such as pain, vomiting, loss of appetite, ascites, jaundice, constipation, and diarrhea, among patients with liver cancer. Moreover, the continuous scores on PG-SGA can prioritize the treatments that are more needed by patients, thus allowing for a more efficient use of resources. However, the use of PG-SGA requires evaluators to be systematically trained and have certain experience, which takes a relatively long time, posing an obstacle to medical staff with heavy daily work tasks.

The results of this study showed that age, tumor diameter, TNM stage, and anemia are independent influencing factors for the short-term nutritional risk of patients with hepatocellular carcinoma. Specifically, those aged > 51 years had a higher risk of nutritional risk, which may be attributed to the decline of liver function, and the combination of chronic diseases, which increases the likelihood of malnutrition. Furthermore, larger tumor diameters, late TNM staging, and anemia were associated with a 37.678-fold, 7.642-fold, and 6.663-fold increased risk of nutritional risk, respectively. These factors may affect the patient's need to consume more energy and protein, and the resulting obstruction and bleeding may easily damage the digestion and absorption functions of the stomach and other digestive organs, thereby affecting tumor progression.

From this study, it can be seen that the AUC of the simplified model, NRS-2002, and PG-SGA for predicting the nutritional risk of patients are different. The AUC of the simplified model was found to be 0.986 [95%CI (0.961,0.997)]. This efficacy criterion of AUC > 0.9 is considered excellent and is more advantageous than NRS-2002 and PG-SGA alone in identifying patients with higher nutritional risk in hepatocellular carcinoma patients. This score includes age, tumor diameter, TNM stage, and whether or not anemia as variables, which are relatively easy to collect, allowing nurses to quickly evaluate patients in their daily work. Patients with a score > 4 points are suggested to have a higher nutritional risk. Rapid assessment with the use of NRS-2002 and PG-SGA followed by timely nutritional intervention for patients can effectively reduce the morbidity and mortality of nutrition-related diseases, thereby improving the quality of life of patients.

## Conclusion

In this study, a short-term nutritional risk prediction model for hepatocellular carcinoma patients was established by utilizing four factors: age, tumor diameter, TNM stage, and anemia. The use of the prediction model has more advantages since the data is easy to obtain and requires no additional training, which is suitable for nursing staff to use in the rapid assessment of nutritional risk in patients with hepatocellular carcinoma.

## Limitations

This study, while offering significant insights into the nutritional risk in hepatocellular carcinoma patients, is subject to certain limitations. Primarily, its retrospective nature inherently limits our ability to establish causal relationships between variables and nutritional risk. Additionally, the cohort consisted exclusively of patients with isolated hepatocellular carcinoma and less severe liver impairment, excluding those with advanced disease. This selective criteria means that the findings might not be applicable to all hepatocellular carcinoma patients, particularly those with more advanced stages of the disease.

Moreover, the DXA modality utilized in our study primarily assessed body composition, specifically muscle and fat mass, while it did not provide direct measurements of other pivotal factors associated with malnutrition, such as dietary intake, nutrient absorption, or functional impairment. Secondly, DXA-derived results may be susceptible to fluctuations in body water content, notably observed in patients with liver disease, potentially introducing variability in the precision of DXA interpretations. Additionally, the applicability of DXA reference values and cutoffs may exhibit variations contingent upon factors such as race and age, posing the risk of misclassifying nutritional status when inappropriate references are employed.

The study was conducted over a specific time frame (March 2021–April 2022), which may limit the generalizability of the findings to different periods, especially considering potential changes in treatment protocols or patient demographics. The reliance on data from a single medical institution might not accurately represent the broader hepatocellular carcinoma patient population. While the model incorporated factors such as age, tumor diameter, TNM stage, and anemia, it's possible that other relevant variables influencing nutritional risk were not included in the study. Lastly, the model's short-term focus may not fully capture the long-term nutritional risks and outcomes for these patients, underscoring the need for further longitudinal research.

## Data Availability

The data underlying this article will be shared on reasonable request to the corresponding author.
