# Author Correction: Development of a short-term nutritional risk prediction model for hepatocellular carcinoma patients: a retrospective cohort study

**DOI:** 10.1038/s41598-024-65133-x

**Published:** 2024-06-19

**Authors:** Jiaxiang Yu, Kim Lam Soh, Liping He, Pengpeng Wang, Yingjuan Cao

**Affiliations:** 1 Department of Nursing, Universiti Putra Malaysia, 43400 Serdang, Selangor Malaysia; 2 Nursing Department, Qilu Hospital, Shandong University, Jinan, 250012 China

Correction to: *Scientific Reports* 10.1038/s41598-024-54456-4, published online 16 February 2024

The original version of this Article contained an error in the name of author Kim Lam Soh, which was incorrectly given as Soh Kim Lam.

The original Article has been corrected.

